# Research on Blind Super-Resolution Technology for Infrared Images of Power Equipment Based on Compressed Sensing Theory

**DOI:** 10.3390/s21124109

**Published:** 2021-06-15

**Authors:** Yan Wang, Lingjie Wang, Bingcong Liu, Hongshan Zhao

**Affiliations:** School of Electrical & Electronic Engineering, North China Electric Power University, Baoding 071003, China; wang_yan0421@163.com (Y.W.); sas_liubingcong@163.com (B.L.); zhaohshcn@ncepu.edu.cn (H.Z.)

**Keywords:** power equipment, infrared image, compressed sensing, blind super-resolution

## Abstract

Infrared images of power equipment play an important role in power equipment status monitoring and fault identification. Aiming to resolve the problems of low resolution and insufficient clarity in the application of infrared images, we propose a blind super-resolution algorithm based on the theory of compressed sensing. It includes an improved blur kernel estimation method combined with compressed sensing theory and an improved infrared image super-resolution reconstruction algorithm based on block compressed sensing theory. In the blur kernel estimation method, we propose a blur kernel estimation algorithm under the compressed sensing framework to realize the estimation of the blur kernel from low-resolution images. In the estimation process, we define a new $L_{w}$ norm to constrain the gradient image in the iterative process by analyzing the significant edge intensity changes before and after the image is blurred. With the $L_{w}$ norm, the salient edges can be selected and enhanced, the intermediate latent image generated by the iteration can move closer to the clear image, and the accuracy of the blur kernel estimation can be improved. For the super-resolution reconstruction algorithm, we introduce a blur matrix and a regular total variation term into the traditional compressed sensing model and design a two-step total variation sparse iteration (TwTVSI) algorithm. Therefore, while ensuring the computational efficiency, the boundary effect caused by the block processing inside the image is removed. In addition, the design of the TwTVSI algorithm can effectively process the super-resolution model of compressed sensing with a sparse dictionary, thereby breaking through the reconstruction performance limitation of the traditional regularized super-resolution method of compressed sensing due to the lack of sparseness in the signal transform domain. The final experimental results also verify the effectiveness of our blind super-resolution algorithm.

## 1. Introduction

Infrared thermal imaging technology is an important technical means to ensure the safe and reliable operation of power equipment [1,2]. With the advent of the Internet of Things era and the application of big data, 5G communications, cloud computing and other technologies, online monitoring of equipment through infrared sensors has become an inevitable trend for the future development of power grids. The infrared image of the equipment collected by the sensor is transmitted to the control center and database, and various intelligent algorithms are based on this to accurately evaluate the operating status of the equipment. Large-scale installation of infrared sensors will bring many problems, such as data storage, installation costs, and so on. This makes it difficult to generally install high-precision infrared sensors in the power grid. However, the temperature change speed of power equipment is much slower than the change speed of its electrical parameters, so its infrared image collection frequency does not need to be too high, and its collection period can reach several minutes. A sufficiently long acquisition period provides an opportunity for real-time processing of infrared images through background algorithms. We can use background algorithms to improve the quality of infrared images within a collection interval of several minutes, where the reconstructed result image is used for display and analysis, and the original image is used for storage. This mode of collecting low-quality images on-site and reconstructing high-quality images in the background can greatly reduce the cost of sensor installation and reduce the pressure of data storage. The super-resolution technology that has emerged in recent years has provided us with new ideas for establishing the above-mentioned model.

Super-resolution (SR) aims to reconstruct a high-quality image x from its degraded measurement y [3]. SR is a typical ill-posed inverse problem and it can be generally modelled as

(1) y=(h⊗x)↓+η

where h is a blur kernel, ⊗ denotes the convolution operator, and ↓ the down-sampling operator. According to the number of input images, SR technology is divided into single image super-resolution (SISR) technology and multiframe image super-resolution (MISR) technology. SISR technology is relatively difficult to implement due to the limited original information. However, an excessively high image acquisition frequency will put great pressure on the data transmission channel and storage space. Therefore, the infrared image acquisition cycle of power equipment is generally longer, thus making it impossible to continuously obtain multiple infrared images of the same power equipment in a very short time. As the conditions for adopting MISR technology are not available, the focus of this paper is on SISR technology. The problem is inherently ill-posed as a single low-resolution image can map to several high-resolution images. In the existing technology to solve this problem, interpolation is the most classic reconstruction method, including bilinear, bicubic, and spline interpolation methods. The principle of the interpolation method is simple and the amount of calculation is small, but the reconstructed image will have problems such as loss of detail and blurred texture. In order to resolve the above problems, many improved interpolation methods have been proposed, such as displacement field interpolation, wavelet theory interpolation, and regression model interpolation [4,5,6]. In addition to interpolation methods, with the development of artificial intelligence technology in recent years, image super-resolution methods based on sample learning have become a research hotspot. In 2002, Freeman [7] proposed obtaining the mapping relationship between LR images and HR images through machine learning so as to maximize the posterior probability of HR images when the LR image sequence is known. In 2010, Yang et al. [8] proposed the acquisition of a sparse isomorphic high/low-resolution dictionary through joint training of LR images and HR images. Therefore, the corresponding high-resolution image block can be obtained by inputting the low-resolution image block. Since then, a large number of super-resolution algorithms based on sample learning have emerged [9,10]. The learning-based super-resolution method has a strong ability to restore image information, but a large number of high-definition images and high-performance hardware equipment support are required in the early model training. In addition, the reconstructed HR image may have false textures. This defect will adversely affect operations such as image segmentation, target recognition, and fault location in the process of infrared monitoring and diagnosis. Therefore, researchers still need to overcome certain difficulties in applying learning-based super-resolution technology to infrared images of power equipment. In addition to interpolation methods and learning methods, reconstruction-based methods are also a common type of SR method. Pejman et al. [11] proposed an iterative back-projection method, which calculates and compensates for the difference between a low-resolution image and an observed image to obtain a high-resolution reconstructed image. X. Zhang et al. [12] proposed a method to establish a nonlocal total variation model based on the nonlocal self-similarity of the image to achieve super-resolution. In order to better process the local information of an image, Li et al. [13] used the self-similarity of the local structure in natural images and proposed the concept of turning to kernel regression total variation. Then, the nonlocal regular total variation term is used as a supplement to form its super-resolution reconstruction model. Although this type of SR method based on internal self-similarity of images can often achieve better application effects when processing images with repeated information, it has poor performance when applied to images without self-similar structures. In [14], the authors proposed a SR method based on Tikhonov regularization. However, because the contour of the reconstruction result was too smooth, the researchers further improved it. For example, Huo et al. [15] introduced the TV regularization term into the Tikhonov regularization model, and achieved better results by adjusting the intensity of different regularization terms through adaptive control. In addition, the method based on maximum a posteriori probability (MAP) has also achieved good application effects in the field of super-resolution by introducing different prior information to the model. For example, Liu et al. [16] improved both the fidelity and regularization terms of the model based on the MAP theory. They used semiquadratic estimation to adaptively select the error norm for the fidelity term, and proposed a new regularization method based on adaptive bilateral total variation for the prior information. This method has achieved good results in denoising and edge protection. In addition to the above methods, because compressed sensing (CS) can accurately reconstruct signals at a sampling frequency much lower than that for Nyquist’s sampling law, it has been initially applied in the field of SR. However, as its theoretical application is restricted by sampling conditions, there are few regularized compressed sensing SR methods. At present, SR research based on compressed sensing focuses on the construction of isomorphic sparse dictionaries. Through the establishment of a high- and low-resolution image symbiosis model, detailed information is “added” to the LR image to be reconstructed. Although this method has a better visual effect, it also requires a lot of HR images for pretraining, and the problem of false texture is unavoidable.

However, most of the above-mentioned super-resolution methods assume that there is no blur in the imaging process or that the blur kernel is known. According to the actual imaging model, it is known that the above two assumptions are not reasonable. In actual situations, blur often exists, and the blur kernel is generally unknown, or only partial prior information is known [17,18]. Therefore, in the SR reconstruction process, it is necessary to estimate the blur kernel through the existing LR image so that the performance loss of the SR method can be reduced. This type of method is known as blind super-resolution, and the research on this is relatively limited. Shao et al. [19] proposed an adaptive heavy-tail image prior, which is a normalized sparseness measure and a generalized integral of the relative total variation. Finally, the high-resolution image and blur kernel are estimated through alternate iterations. Qian et al. [20] first deblurred a low-resolution image and then used the weighted cost function for SR to obtain a high-resolution image. Kim et al. [21] proposed a low-complexity adaptive SISR method. Based on the relationship between the input image and its low-resolution version, this method adaptively determines the intensity of the high-frequency components to be added, thereby estimating the missing high-frequency details in the upsampled image to improve the quality. Michaeli et al. [17] used the recursion of similar structures in an image to realize the estimation of the blur kernel. Most blind super-resolution methods are based on accurate estimation of the blur kernel. According to the existing research, accurate estimation of the blur kernel largely depends on the selection of the significant edges of the image, which is reflected in many blind deblurring methods [22,23,24]. However, although the extraction of salient edges has attracted the attention of researchers, the existing methods have not paid attention to the changes in the distribution of salient edges before and after the image is blurred and used.

Based on the above discussion, we propose a maximum a posterior (MAP)-based blind super-resolution algorithm by using the CS theory. First, the basic CS super-resolution model is combined with the blind image deblurring theory, and a blur kernel estimation algorithm under the CS framework is proposed, thereby realizing the estimation of the blur kernel from the low-resolution image. In the estimation process, through the analysis of the significant edge intensity changes before and after the image is blurred, a new $L_{w}$ norm is defined to constrain the gradient image in the iterative process. In this way, the salient edges can be selected and enhanced, the intermediate latent image generated by the iteration can move closer to the clear image, and the accuracy of the blur kernel estimation can be improved. After completing the blur kernel estimation, a corresponding blur matrix is generated, and the blur matrix is added to the CS original model to construct a CS super-resolution model based on the principle of image degradation. The sparse representation of the infrared image in the model is realized by a specific sparse dictionary about the infrared image of the power equipment obtained using the high-dimensional dictionary training method. Finally, in the SR reconstruction process, in order to improve the computational efficiency, the block compressed sensing (BCS) method is used to reconstruct the LR infrared image by blocks. Aiming to resolve the “blocking effect” problem in the BCS method, a two-step total variation sparse iteration (TwTVSI) algorithm is designed. While ensuring the computational efficiency, the boundary effect caused by the block in the image is removed and the design of the TwTVSI algorithm can effectively process the CS super-resolution model with a sparse dictionary, thus breaking through the reconstruction performance limitation of the traditional regularized CS super-resolution method due to the lack of sparsity in the signal transform domain. The final experimental results also verify the effectiveness of our blind super-resolution algorithm.

## 2. CS Blind SR Model Based on the Principle of Image Degradation

As a brand-new signal sampling theory, CS enables signal reconstruction to break through the Nyquist sampling law and greatly reduces the number of sampling points required for accurate signal recovery [25]. The basic model is as follows:

(2) y=Φx

where $x\in R^{n}$ is a high-dimensional signal; Φ is a sampling matrix; and $y\in R^{m}$. is a low-dimensional sampling signal, n≫m. If the signal x is a sparse signal, its sparsity k satisfies k>2m, and the sampling matrix Φ satisfies the restricted isometry property (RIP) criterion [26], the signal can be accurately reconstructed.

The image is recorded as a two-dimensional signal X, which can also be converted into a one-dimensional signal x after expanding its pixel points by column. Although the image signal x is not sparse in the spatial domain, the sparse transformation matrix Ψ can be added to perform sparse transformation on x when the CS theory is used to reconstruct the signal; therefore, the model can be as follows:

(3) $$y=\Phi \Psi \tilde{x}=A\tilde{x}$$

where $\tilde{x}$ is a sparse signal, $x=\Psi \tilde{x}$. The matrix Ψ can be an artificially constructed over-complete dictionary or a sparse basis such as Fourier basis, cosine basis, wavelet basis, etc. A=ΦΨ is the sensing matrix; if A satisfies the RIP criterion, the sparse signal $\tilde{x}$ can be reconstructed by y, and x can be obtained by calculating $\Psi \tilde{x}$.

Considering the cost of sensors and the pressure of data transmission, it is difficult to directly obtain clear, HR infrared images for condition monitoring and fault diagnosis of power equipment. Therefore, it is necessary to use SR technology on the LR images collected by low-precision sensors. Then, the input signal y will be a low-resolution image signal, not a random sampling signal, so the sampling matrix Φ cannot use random sampling matrices such as random Gaussian matrices and random Bernoulli matrices that have been proven to be universal. Φ needs to be constructed according to the image degradation process, and the image degradation model is as follows:

(4) Y=(h⊗X)↓+η

where Y is the LR image; X is the HR image; h is the blur kernel; ⊗ denotes the convolution operation; ↓ is the downsampling process, and its corresponding sampling matrix Φ is mostly a cubic interpolation downsampling matrix or point sampling matrix; and η is the noise.

It can be seen from the image degradation model that Φ is a nonrandom sampling matrix, and its corresponding sensing matrix A=ΦΨ has a low degree of satisfaction with the RIP criterion. When directly using the Formula (4) model for super-resolution reconstruction, the visual quality of the reconstructed image is poor. In order to improve the quality of image reconstruction, we introduce the principle of image degradation into the compressed sensing model to bring the model closer to the actual image acquisition process and thus obtain the following:

(5) $$y=CHD\tilde{x}$$

where C is the sampling matrix; H is the blur matrix constructed according to the blur kernel, h; and D is the sparse dictionary. The construction method of H is introduced in Section 3. At this time, the sensing matrix A=CHD. After experimental verification, the blur matrix is added to the model, which enables deconvolution and deblurring during SR reconstruction of the image, thus achieving the effect of sharpening high-resolution images and enhancing their detailed edges and contours. This is proven in Section 5.

## 3. Blur Kernel Estimation and Construction of Blur Matrix

### 3.1. Blur Kernel Estimation

Since the blurring process is common during image acquisition and is generally unknown [17,18], it is necessary to estimate the blur kernel h according to the collected LR infrared images so as to construct an accurate blur matrix H. Therefore, we designed a blur kernel estimation method based on the principle of compressed sensing and prior knowledge of image gradient to obtain an accurate h.

#### 3.1.1. Blur Kernel Estimation Model Based on Image Gradient Prior

The extraction effect of the significant edges of an image is directly related to the accuracy of the blur kernel estimation. The current common methods mostly use the $L_{0}$ norm to constrain the image gradient to obtain the significant edge that is conducive to the blur kernel estimation [24,27,28,29]. However, these methods all aim to remove the small edges that hinder the estimation of the blur kernel and do not consider the changing law of the edge distribution before and after the image is blurred. Based on prior knowledge of the gradient change rule before and after the image is blurred, we designed corresponding constraints to promote the intermediate latent image in the iterative process to move closer to the clear image, thereby improving the accuracy of the blur kernel estimation.

**Figure 1:**
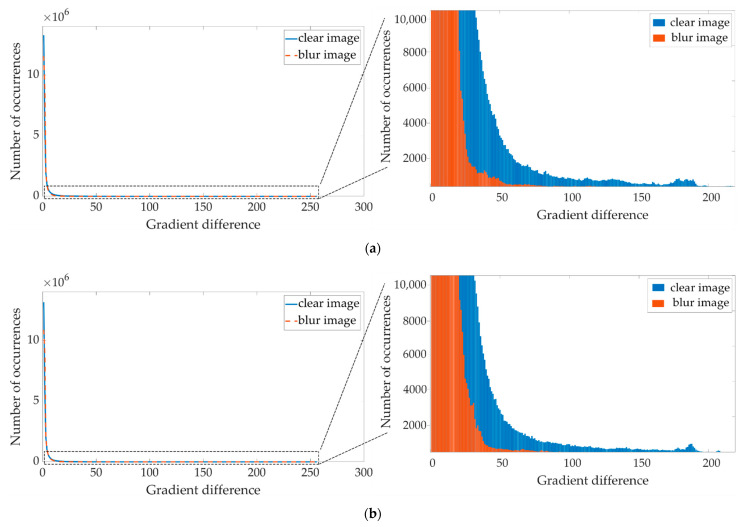
Gradient contrast map of infrared images before and after blurring: (**a**) row direction gradient contrast of clear and blurred images; (**b**) column direction gradient contrast of clear and blurred images.

The gradient distribution of an infrared image has strong sparsity both before and after blurring. However, we noticed that the gradient value of the image generally shows a decreasing trend in the process of blurring, which is especially obvious in the part of the large gradient value. Through the gradient operators [−1,1] and ${\left[-1,1\right]}^{T}$ convolving 500 infrared images of power equipment before and after blurring, we obtained the distribution of the absolute values of the gradient of the clear and blurred images in the row direction and column direction, respectively, as shown in Figure 1a,b.

**Figure 2:**
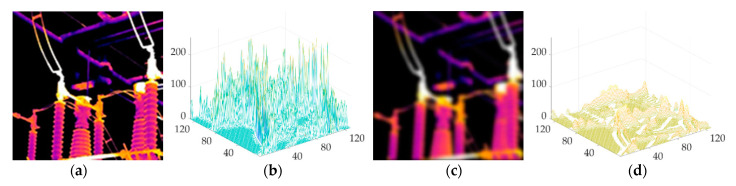
Schematic diagram of gradient changes before and after the infrared image is blurred: (**a**) clear infrared image; (**b**) absolute value of the row direction gradient of the clear infrared image; (**c**) blurred infrared image; (**d**) absolute value of the gradient in the row direction of the blurred infrared image.

In simple terms, this phenomenon is caused by the weighted summation of pixel values that occur in the blurring process. From Figure 2a,c, it is also obvious that for a certain device image, after the infrared image is blurred, the edge contour is no longer clear, which is reflected in the gradient value—i.e., its distribution is closer to zero. According to the statistics of the absolute value of the gradient in the row direction of the grayscale image, there are many large gradient values exceeding 100, as shown in Figure 2b, and after blurring, the overall gradient value is distributed below 100, as shown in Figure 2d. This also causes the significant edges corresponding to the large gradient to be significantly weakened, which adversely affects the estimation of the blur kernel.

In order to eliminate the small gradients while enhancing the significant edges of the blurred infrared image so that the intermediate latent image in the reconstruction process can move closer to the clear infrared image, and to improve the accuracy of the blur kernel estimation, a new calculation method was defined as follows:

(6) $$\hat{X}=\arg\underset{X}{\min}\alpha_{1}{\left\|X-B\right\|}_{2}^{2}+\alpha_{2}{\left\|X\right\|}_{w}$$

(7) $${\left\|X\right\|}_{w}=\overset{M}{\underset{m=1}{\sum }}\overset{N}{\underset{n=1}{\sum }}W\left(X_{m,n}\right)$$

(8) $$W\left(X_{m,n}\right)=\{\begin{array}{cc} -\left|X_{m,n}\right|, & \left|B_{m,n}\right|>\frac{\alpha_{2}}{2\alpha_{1}}\\ \left|X_{m,n}\right|, & otherwise \end{array}$$

where m and n are the row and column coordinates of the matrix, respectively; M and N represent the number of rows and columns of the matrix, respectively; $\sigma_{1}\;\mathrm{and}\;\sigma_{2}$ are weight coefficients; ${\left\|\cdot \right\|}_{w}$ is called the $L_{w}$ norm, and its operation method is shown in Formulas (6)–(8). We use the $L_{w}$ norm instead of the $L_{0}$ norm to constrain the image gradient to extract and enhance the significant edges of the image in the process of blur kernel estimation. 

According to the MAP theory, the blur kernel estimation objective function can be expressed as:

(9) $$\hat{x},\hat{h}=\arg\underset{x,h}{\min}{\left\|y-Ax\right\|}_{2}^{2}+\upsilon_{x}g_{x}\left(x\right)+\upsilon_{h}g_{h}\left(h\right)$$

where ${\left\|y-Ax\right\|}_{2}^{2}$ is the data fidelity item; $g_{x}\left(x\right)$ is the regular term introduced based on the prior information of the image, $g_{h}\left(h\right)$ is the regular term introduced based on the prior information of the blur kernel. The specific model of blur kernel estimation can be obtained:

(10) $$\left(\hat{X},\hat{h}\right)=\arg\underset{X,h}{\min}\|Y-S_{l}\left(h⊗X\right)S_{r}\|{}_{2}^{2}+\lambda \|\nabla X\|{}_{w}+\gamma {\left\|h\right\|}_{2}^{2}+\mu \|D{\left(X\right)\|}_{0}+\delta \|\Psi^{T}X\|{}_{1}$$

where λ,μ,δ,and γ are weighting coefficients. The first term of the equation is the data fidelity term, which is used to ensure that there is a correspondence between the intermediate latent image X generated by the iteration and the LR blurred image Y. The data difference of this item can be constrained by the $L_{2}$ or $L_{1}$ norm [30]; here, we use the $L_{2}$ norm as the data fidelity function. The second term is the constraint term for significant gradient extraction and enhancement. The third term is the constraint adopted for the sparseness of the blur kernel. The fourth item is a dark channel prior (DCP) constraint item, where $D\left(X\right)\left(p\right)=\underset{q\in N\left(p\right)}{\min}\left(\underset{c\in \left\{r,g,b\right\}}{\min}X^{c}\left(q\right)\right)$, and $X^{c}$ is a color channel of c. If the image is a single channel such as grayscale or brightness, then $\underset{c\in \left\{r,g,b\right\}}{\min}X^{c}\left(q\right)=X\left(q\right)$, from which we observe that DCP is used to represent the minimum value in the image block. It can be seen from Formula (4) that the blurring process can be regarded as the local weighted linear summation of the clear image. Therefore, the weighted sum of pixel values in a local neighborhood is larger than the minimum pixel value in the neighborhood, i.e., blur increases the values of the dark pixels. As such, the dark channel of blurred images is less sparse than that of the clear images. The change in the sparsity of the dark channel is an inherent characteristic of the blurring process. Based on this, by minimizing $\|D{\left(X)\right\|}_{0}$, our model will tend toward clear images rather than blurred images during iteration. This constraint can also effectively improve the estimation accuracy of the blur kernel [28]. The last item is used to ensure that the sparse coefficients obtained after the sparse transformation of the image are sparse enough and is combined with the first item to achieve high-resolution intermediate latent images.

In addition, in order to ensure the calculation efficiency of the blur kernel estimation, we did not stretch the image into a column vector in the original compressed sensing before the calculation. The reason is that if the image is not divided into blocks but directly stretched as a column vector for calculation, the downsampling matrix will be too large and the calculation speed will be greatly reduced. If it is divided into blocks, corresponding boundary problems will be introduced. Therefore, we modified the original model slightly, constructing a row sampling matrix $S_{l}$ and a column sampling matrix $S_{r}$ according to the principle of cubic interpolation so that the downsampling operation is performed twice. The size of the downsampling matrix and the position of nonzero elements are determined according to the downsampling rate, and the element values are determined according to the cubic interpolation downsampling function.

#### 3.1.2. Solving Subproblem X

In order to solve Equation (10) quickly, our strategy was to adopt the half quadratic splitting algorithm [31] and the alternate minimization method for calculation. Firstly, the variable G is introduced through the half quadratic splitting method. Then, (10) can be transformed into (11):

(11) $$\begin{array}{l} \left(X^{\prime },h^{\prime }\right)=\arg\underset{X,h}{\min}\|Y-S_{l}GS_{r}\|{}_{2}^{2}+\lambda \|\nabla X\|{}_{w}+\gamma \|h\|{}_{2}^{2}+\mu \|D{\left(X\right)\|}_{0}\\ \;\;\;\;\;\;\;\;\;\;\;\;\;\;+\delta \|\Psi^{T}X\|{}_{1}+\varepsilon \|h⨂X-G\|{}_{2}^{2} \end{array}$$

The solution of X can then be obtained by solving

(12) $$\left(X^{\prime },h^{\prime }\right)=\arg\underset{X,h}{\min}\lambda \|\nabla X\|{}_{w}+\mu \|D\left(X)\right\|{}_{0}+\delta \|\Psi^{T}X\|{}_{1}+\varepsilon \|h⨂X-G\|{}_{2}^{2}$$

Since the model (12) contains the $L_{0}$ norm and the $L_{1}$ norm, it is difficult to directly solve the variables by means of fast Fourier transform. We introduce auxiliary variables g,d and $\tilde{X}$, corresponding to ∇X,D(X) and $\Psi^{T}X$ respectively, where $g=\left\{g_{h},g_{v}\right\}$, $\nabla X=\left\{\nabla_{h}X,\nabla_{v}X\right\}$. $\nabla_{h}X\;\mathrm{and}\;\nabla_{v}X$ are row difference images and column difference images, respectively, and the difference operators are defined as $\nabla_{h}=\left[-1,1\right],\nabla_{v}={\left[-1,1\right]}^{T}$. Then, (12) can be transformed into (13):

(13) $$\begin{array}{l} \left(X^{\prime },h^{\prime },g^{\prime },d^{\prime },{\tilde{X}}^{\prime }\right)=\arg\underset{X,h,g,d,\tilde{X}}{\min}\lambda^{\prime }\|g-\nabla {X\|}_{2}^{2}+\mu^{\prime }\|d-D{\left(X)\right\|}_{2}^{2}++\delta^{\prime }\|\tilde{X}-\Psi^{T}{X\|}_{2}^{2}\\ \;\;\;\;\;\;\;\;\;\;\;\;\;\;\;\;\;\;\;\;\;\;\;\;\;\;\;\;\;\;\;\;+\lambda \|{g\|}_{w}+\mu \|{d\|}_{0}+\delta \|{\tilde{X}\|}_{1}+\varepsilon \|h⨂X-{G\|}_{2}^{2} \end{array}$$

where $\lambda^{\prime },\;\mu^{\prime },\;\mathrm{and}\;\delta^{\prime }$ are the regularization parameters. The above optimization problem can be solved by minimizing X,h,g, and d and $\tilde{X}$, respectively. When a variable is minimized, the other variables are regarded as fixed. Fixing h,g, and d and $\tilde{X}$, the solution of X can be obtained by solving the following:

(14) $$X^{\prime }=\arg\underset{X}{\min}\lambda^{\prime }\|g-\nabla {X\|}_{2}^{2}+\mu^{\prime }\|d-D{\left(X\right)\|}_{2}^{2}++\delta^{\prime }\|\tilde{X}-\Psi^{T}{X\|}_{2}^{2}+\varepsilon \|h⨂X-{G\|}_{2}^{2}$$

where $\|g-\nabla {X\|}_{2}^{2}=\|g_{h}-\nabla_{h}{X\|}_{2}^{2}+\|g_{v}-\nabla_{v}{X\|}_{2}^{2}$. The solution of (14) can be obtained by using the fast Fourier transform:

(15) $$X=F^{-1}\left(\frac{\lambda^{\prime }F_{g}+\mu^{\prime }F\left(M^{T}d\right)+\delta^{\prime }F\left(\Psi \tilde{X}\right)+\varepsilon \bar{F\left(h\right)}\circ F\left(G\right)}{\lambda^{\prime }F_{\nabla }+\mu^{\prime }+\delta^{\prime }+\varepsilon \bar{F\left(h\right)}\circ F\left(h\right)}\right)$$

where $F_{g}$ is $\bar{F\left(\nabla_{h}\right)}\circ F\left(g_{h}\right)+\bar{F\left(\nabla_{v}\right)}\circ F\left(g_{v}\right)$; $F_{\nabla }$ is $\bar{F\left(\nabla_{h}\right)}\circ F\left(\nabla_{h}\right)+\bar{F\left(\nabla_{v}\right)}\circ F\left(\nabla_{v}\right)$; F(⋅) and $F^{-1}\left(\cdot \right)$ denote the fast Fourier transform and inverse fast Fourier transform, respectively; $\bar{F\left(\cdot \right)}$ is the complex conjugate operator; ∘ denotes component multiplication, and the division in Formula (15) is component division; M is the matrix that maps the image to its dark channel; and $M^{T}$ is the transpose of M, which specifies that it plays the role of the reverse mapping replacement [28]. The mapping method of the M matrix is determined by the index between the image and its dark channel:

(16) $$M\left(p,q\right)=\{\begin{array}{cc} 1, & q=\arg\underset{q\in N\left(p\right)}{\min}X\left(q\right)\\ 0, & otherwise \end{array}$$

After the calculation of X, fixing $h,g,\tilde{X}$, and X, the solutions of d can be obtained by solving the following:

(17) $$d^{\prime }=\arg\underset{d}{\min}\mu^{\prime }\|d-D{\left(X\right)\|}_{2}^{2}+\mu \|{d\|}_{0}$$

Shrinking by hard threshold, the solutions of d can be obtained based on (18):

(18) $$d=\{\begin{array}{cc} D\left(X\right), & {\left(D\left(X\right)\right)}^{2}\geq \frac{\mu }{\mu^{\prime }}\\ 0, & otherwise \end{array}$$

Fixing h,g,d, and X, the solutions of $\tilde{X}$ can be obtained by solving the following:

(19) $${\tilde{X}}^{'}=\arg\underset{\tilde{X}}{\min}\delta^{\prime }\|\tilde{X}-\Psi^{T}{X\|}_{2}^{2}+\delta \|{\tilde{X}\|}_{1}$$

Shrinking by soft threshold, the solutions of $\tilde{X}$ can be obtained based on (20):

(20) $$\tilde{X}=\max\left\{\left|\Psi^{T}X\right|-\frac{\delta }{2\delta^{\prime }},0\right\}\circ \mathrm{sign}\left(\Psi^{T}X\right)$$

where sign(⋅) is a sign function. The subproblem with g can be solved by the following:

(21) $$g^{\prime }=\arg\underset{g}{\min}\lambda^{\prime }\|g-\nabla {X\|}_{2}^{2}+\lambda \|{g\|}_{w}$$

According to the definition of the $L_{w}$ norm, the solution of Equation (21) is easily obtained:

(22) $$g=\{\begin{array}{cc} \nabla X+\frac{\lambda }{2\lambda^{\prime }}, & \nabla X>\frac{\lambda }{2\lambda^{\prime }}\\ 0, & otherwise\\ \nabla X-\frac{\lambda }{2\lambda^{\prime }}, & \nabla X<-\frac{\lambda }{2\lambda^{\prime }} \end{array}$$

The solution result shows that by using the $L_{w}$ norm to constrain the gradient image, it is possible to filter out smaller gradients and enhance significant gradients. In addition to the above variables, the variable G is also solved in the X subproblem; that is, in the h subproblem, the variable G is fixed. The solution of G can be obtained by solving the following:

(23) $$G^{\prime }=\arg\underset{G}{\min}\|Y-S_{l}GS_{r}\|{}_{2}^{2}+\varepsilon \|h⊗X-{G\|}_{2}^{2}$$

which is a least squares minimization problem; we compute the solution using the gradient descent method. The iteration step size in the solution process is determined by the non-monotonic linear search method introduced in [32].

#### 3.1.3. Solving Subproblem h

For the subproblem h, directly using the intermediate latent image to estimate the blur kernel is not accurate [30]; therefore, the gradient image is used to estimate the blur kernel. Then, the solution of h can be obtained by solving the following:

(24) $$h^{\prime }=\arg\underset{h}{\min}\varepsilon \|h⊗\nabla X-\nabla {G\|}_{2}^{2}+\gamma \|{h\|}_{2}^{2}$$

which is a least squares minimization problem. Similar to the existing approaches [29,30,33], we compute the solution using FFT:

(25) $$h=F^{-1}\left(\frac{\bar{F\left(\nabla X\right)}F\left(\nabla G\right)}{\bar{F\left(\nabla X\right)}F\left(\nabla X\right)+\gamma }\right)$$

Since the blur kernel h≥0 and $\|{h\|}_{1}=1$, after each iteration of the h subproblem, we set the negative elements of h to zero and normalize h at the end. Algorithm 1 shows the main steps for the kernel estimation algorithm. As suggested by [24,30], we decrease λ and μ gradually to make more information available for kernel estimation.


**Algorithm 1:** Blur Kernel Estimation Algorithm.
Input: Blurred image Ygenerate the initial value of each variablefor i=1:5 do

$\varepsilon \leftarrow 2\varepsilon_{0}$
 repeat solve for G using the gradient descent method, $\mu^{\prime }\leftarrow 2\mu$.repeatsolve for d using (18), $\delta^{\prime }\leftarrow 2\delta$.repeatsolve for $\tilde{X}$ using (20), $\lambda^{\prime }\leftarrow 2\lambda$.repeatsolve for g using (22), solve for X using (15), $\lambda^{\prime }\leftarrow 2\lambda^{\prime }$.until $\lambda^{\prime }>\lambda_{max}^{\prime }$$\delta^{\prime }\leftarrow 2\delta^{\prime }$.until $\delta^{\prime }>\delta_{max}^{\prime }$$\mu^{\prime }\leftarrow 2\mu^{\prime }$.until $\mu^{\prime }>\mu_{max}^{’}$ε←2ε. until $\varepsilon >\varepsilon_{max}$ solve for h using (25). λ←0.9λ, μ←0.9μ.end forOutput: blur kernel h.


### 3.2. Blur Matrix Construction

In order to facilitate the operation of the BCS algorithm, we generated a circular Toeplitz matrix to convert the convolution operation between the blur kernel h and the two-dimensional image signal X into a matrix multiplication operation between the blur matrix H and the one-dimensional image signal x; that is, convert h⊗X to Hx.

Suppose G is a degraded image of X, i.e., G=h⊗X. The size of G is the same as that of X, and both are M×N. Since the blur kernel h is mostly square and the number of columns is odd, its size is recorded as L×L, and l=(L−1)/2 is the radius of h. Then, the discretized degradation model is described as follows:

(26) $$G\left(i,j\right)=\overset{M-1}{\underset{m=0}{\sum }}\overset{N-1}{\underset{n=0}{\sum }}X\left(m,n\right)h\left(i-m+l,j-n+l\right)$$

where h(i,j) needs to be zero-filled and extended as follows:

$h\left(i,j\right)=\{\begin{array}{c} h\left(i,j\right)\;0\leq i\leq L-1\;\mathrm{and}\;0\leq j\leq L-1\\ 0\;L\leq i\leq M-1\;\mathrm{or}\;L\leq j\leq N-1 \end{array}$; we extend the period of X(i,j) and h(i,j) so that they have a period of M in the row direction and a period of N in the column direction.

After the matrices G(i,j) and X(i,j) are arranged from left to right and top to bottom, one-dimensional signals g and x of length MN are obtained. In order to further convert the discretization degradation model of G=h⊗X into Hx, the H with the size of MN×MN can be defined as follows:

(27) $$H=\left[\begin{array}{ccccc} H_{l} & H_{M+l-1} & H_{M+l-2} & \cdots & H_{l+1}\\ H_{l+1} & H_{l} & H_{M+l-1} & \cdots & H_{l+2}\\ H_{l+2} & H_{l+1} & H_{l} & \cdots & H_{l+3}\\ \vdots & \vdots & \vdots & \ddots & \vdots \\ H_{M+l-1} & H_{M+l-2} & H_{M+l-3} & \cdots & H_{l} \end{array}\right]$$

where

$$H_{a}=\left[\begin{array}{ccccc} h\left(a,l\right) & h\left(a,N+l-1\right) & h\left(a,N+l-2\right) & \cdots & h\left(a,l+1\right)\\ h\left(a,l+1\right) & h\left(a,l\right) & h\left(a,N+l-1\right) & \cdots & h\left(a,l+2\right)\\ h\left(a,l+2\right) & h\left(a,l+1\right) & h\left(a,l\right) & \cdots & h\left(a,l+3\right)\\ \vdots & \vdots & \vdots & \ddots & \vdots \\ h\left(a,N+l-1\right) & h\left(a,N+l-2\right) & h\left(a,N+l-3\right) & \cdots & h\left(a,l\right) \end{array}\right]$$

It should be noted that the extension of X and h in the above conversion method is to complement the pixels outside the edge in a cyclic manner. However, this method will introduce large errors to the edge pixels when reconstructing the infrared image of the power equipment by blocks. Therefore, before extending X and h, we must first add zeros to X:

(28) $$X\left(i,j\right)=\{\begin{array}{c} X\left(i-l,j-l\right)\;\;l\leq j\leq N+l-1\\ 0\;\;\;\;\;\;\;\;\;\;\;\;\;\;\;\;\;\;\;\mathrm{otherwise} \end{array}$$

where 0≤i≤M+2l−1 and 0≤j≤N+2l−1. After completing the SR reconstruction of the infrared image according to (5), the reconstructed image can be obtained by deleting the reconstructed pixels at the zero-padded position of the image.

## 4. Image SR Reconstruction Algorithm 

In this paper, the relationship between HR images and LR images proposed by combining CS theory and the image degradation model is shown in (5). In this section, we will establish an SR image reconstruction model based on this relationship and propose an optimized solution algorithm, named two-step total variation sparse iteration (TwTVSI). We introduce the total variation (TV) regular term into the objective function of $L_{1}$ norm reconstruction because the TV regular term can use the limited variation characteristics of the image to eliminate the boundary noise of the BCS reconstructed image. It also prevents the single minimum total variation model from only reflecting the sparse characteristics of the image gradient domain and not making full use of the sparse characteristics of the image change domain, causing problems such as long reconstruction time and poor visual effects.

### 4.1. Objective Function Construction

The original objective function of CS super-resolution based on the principle of image degradation is as follows:

(29) $$\begin{array}{c} \arg\underset{\tilde{x}}{\min}\|{\tilde{x}\|}_{1}\;\\ s.t.\;\;y=CHD\tilde{x} \end{array}$$

As the compressed sensing SR reconstruction of the entire image not only takes up too much storage space but also has a very high computational complexity, we adopted the BCS method to divide the image into several small image blocks and performed SR reconstruction for each small image block separately. We divided the image in the following way:

(30) $$Y=\left(\begin{array}{cccc} Y_{b\left(1,1\right)} & Y_{b\left(1,2\right)} & \cdots & Y_{b\left(1,J\right)}\\ Y_{b\left(2,1\right)} & Y_{b\left(2,2\right)} & \cdots & Y_{b\left(2,J\right)}\\ \vdots & \vdots & \ddots & \vdots \\ Y_{b\left(I,1\right)} & Y_{b\left(I,2\right)} & \cdots & Y_{b\left(I,J\right)} \end{array}\right)$$

where Y is an LR image. $Y_{b\left(i,j\right)}$ is an LR image block; Y is divided into I blocks in the row direction and J blocks in the column direction. $X_{b\left(i,j\right)}$ is the HR image block at the corresponding position of $Y_{b\left(i,j\right)}$, and the size of $X_{b\left(i,j\right)}$ is S×S.

However, the reconstruction method of BCS will introduce a “blocking effect” to the SR image. Therefore, in addition to the sparse prior, we further introduce prior information into Equation (29) to constrain the solution and improve the quality of the reconstructed image based on the MAP theory. Since the image is also sparse in the gradient domain, we introduced the TV regular term in the objective function to perform the minimum variational constraint on the whole image so as to achieve the purpose of eliminating the blocking effect of the reconstructed image. The calculation method of image total variation is as follows:

(31) $$\|{X\|}_{TV}=\underset{i,j}{\sum }\sqrt{{\left(X\left(i,j\right)-X\left(i-1,j\right)\right)}^{2}+{\left(X\left(i,j\right)-X\left(i,j-1\right)\right)}^{2}}$$

where X(i,j) denotes the value contained in the i-th row and j-th column of the image matrix X.

We blocked the image and introduced the TV regular term. The objective function can be rewritten as follows:

(32) $$\begin{array}{c} \arg\underset{\tilde{X}}{\min\;\lambda }\|{X\|}_{TV}+\overset{I}{\underset{i=1}{\sum }}\overset{J}{\underset{j=1}{\sum }}\|{\tilde{X}}_{\left(i,j\right)}\|{}_{1}\;\\ s.t.\;\;y_{\left(i,j\right)}=CHD{\tilde{X}}_{\left(i,j\right)},\;\;\;i=1,2,\ldots I,\;\;\;j=1,2,\ldots ,J \end{array}$$

where ${\tilde{X}}_{\left(i,j\right)}$ is the sparse representation of the column vector obtained by expanding $X_{b\left(i,j\right)}$, and $y_{\left(i,j\right)}$ is the row-expanded column vector corresponding to $Y_{b\left(i,j\right)}$.

In the process of optimizing Equation (32), X is updated under the constraint of $\|{X\|}_{TV}$. When the sparsity constraint is applied to X, it is difficult to obtain ${\tilde{X}}_{\left(i,j\right)}$ from X. This is because D is an over-complete dictionary, so the dimensionality of the image signal is much smaller than the sparse coefficient dimensionality represented by D. In order to facilitate the optimization of the objective function, we used the sparse coefficient matrix $\tilde{X}$ to represent the image matrix X. The representation method is $X=Ar\left(D\tilde{X}\right)$, and the objective function is improved as follows:

(33) $$\begin{array}{c} \arg\underset{\tilde{X}}{\min\;\lambda }\|Ar{\left(D\tilde{X}\right)\|}_{TV}+\overset{I}{\underset{i=1}{\sum }}\overset{J}{\underset{j=1}{\sum }}\|{\tilde{X}}_{\left(i,j\right)}\|{}_{1}\;\\ s.t.\;\;y_{\left(i,j\right)}=CHD{\tilde{X}}_{\left(i,j\right)},\;\;\;i=1,2,\ldots I,\;\;\;j=1,2,\ldots ,J \end{array}$$

where $\tilde{X}=\left({\tilde{X}}_{\left(1,1\right)},{\tilde{X}}_{\left(1,2\right)},\cdots ,{\tilde{X}}_{\left(1,J\right)},{\tilde{X}}_{\left(2,1\right)},\cdots {\tilde{X}}_{\left(I,J\right)}\right)$ is the sparse coefficient matrix. The function Ar(z) denotes that the column vectors in z are spliced into corresponding images in order.

### 4.2. Optimization of Objective Function

Using the Lagrangian multiplier method, (33) can be transformed into (34):

(34) $$\arg\underset{\tilde{X}}{\min\;\lambda }\|Ar{\left(D\tilde{X}\right)\|}_{TV}+\overset{I}{\underset{i=1}{\sum }}\overset{J}{\underset{j=1}{\sum }}\frac{\beta_{\left(i,j\right)}}{2}\|y_{\left(i,j\right)}-CHD{\tilde{X}}_{\left(i,j\right)}\|{}_{2}^{2}+\|{\tilde{X}}_{\left(i,j\right)}\|{}_{1}\;$$

where $\beta_{\left(i,j\right)}$ is the Lagrangian multiplier. For the optimization problem in (34), we propose the TwTVSI optimization algorithm. The iteration format is as follows:

(35) $$\begin{array}{l} {\tilde{X}}^{\left(K+1\right)}={\tilde{X}}^{\left(K\right)}-\mu^{\left(K\right)}\{\lambda \frac{\partial \|Ar{\left(D\tilde{X}\right)\|}_{TV}}{\partial {\tilde{X}}^{\left(K\right)}}+\frac{1}{2}\frac{\partial \sum_{i=1}^{I}\sum_{j=1}^{J}\frac{\beta_{\left(i,j\right)}}{2}\|y_{\left(i,j\right)}-CHD{\tilde{X}}_{\left(i,j\right)}{\|}_{2}^{2}+2\|{\tilde{X}}_{\left(i,j\right)}{\|}_{1}}{\partial {\tilde{X}}^{\left(K\right)}}\}\\ \;\;\;={\tilde{X}}^{\left(K\right)}-\mu^{\left(K\right)}\{\lambda g_{1}\left({\tilde{X}}^{\left(K\right)}\right)+\frac{1}{2}g_{2}\left({\tilde{X}}^{\left(K\right)}\right)\} \end{array}$$

where $\mu^{\left(K\right)}$ is the iteration step size; and $g_{1}\left({\tilde{X}}^{\left(K\right)}\right)$ is the gradient derivative of $\|Ar{\left(D\tilde{X}\right)\|}_{TV}$ to the sparse coefficient matrix ${\tilde{X}}^{\left(K\right)}$, which can be solved by the gradient descent method directly.

(36) $$g_{1}\left({\tilde{X}}^{\left(K\right)}\right)=\frac{\partial \|Ar{\left(D\tilde{X}\right)\|}_{TV}}{\partial {\tilde{X}}^{\left(K\right)}}=\{\frac{\partial \|Ar{\left(D\tilde{X}\right)\|}_{TV}}{\partial {\tilde{X}}_{\left(1,1\right)}^{\left(K\right)}},\ldots ,\frac{\partial \|Ar{\left(D\tilde{X}\right)\|}_{TV}}{\partial {\tilde{X}}_{\left(i,j\right)}^{\left(K\right)}},\ldots ,\frac{\partial \|Ar{\left(D\tilde{X}\right)\|}_{TV}}{\partial {\tilde{X}}_{\left(I,J\right)}^{\left(K\right)}}\}$$

(37) $$\begin{array}{l} \frac{\partial \|Ar{\left(D\tilde{X}\right)\|}_{TV}}{\partial {\tilde{X}}_{\left(i,j\right)}^{\left(K\right)}}=\overset{S}{\underset{m=1}{\sum }}\overset{S}{\underset{n=1}{\sum }}\mathrm{Pd}\left(m,n,{\tilde{X}}_{\left(i,j\right)}^{\left(K\right)},{\tilde{X}}_{\left(i,j\right)}^{\left(K\right)}\right)+\overset{S}{\underset{m=1}{\sum }}\overset{1}{\underset{n=1}{\sum }}\mathrm{Pd}\left(m,n,{\tilde{X}}_{\left(i,j+1\right)}^{\left(K\right)},{\tilde{X}}_{\left(i,j\right)}^{\left(K\right)}\right)\\ \;\;\;\;\;\;\;\;\;\;\;\;\;\;+\overset{1}{\underset{m=1}{\sum }}\overset{S}{\underset{n=1}{\sum }}\mathrm{Pd}\left(m,n,{\tilde{X}}_{\left(i+1,j\right)}^{\left(K\right)},{\tilde{X}}_{\left(i,j\right)}^{\left(K\right)}\right) \end{array}$$

(38) $$\mathrm{Pd}\left(m,n,{\tilde{X}}_{a},{\tilde{X}}_{b}\right)=\frac{\frac{\partial \mathrm{Dr}\left(m,n,{\tilde{X}}_{a}\right)}{\partial {\tilde{X}}_{b}}\times \mathrm{Dr}\left(m,n,{\tilde{X}}_{a}\right)+\frac{\partial \mathrm{Dc}\left(m,n,{\tilde{X}}_{a}\right)}{\partial {\tilde{X}}_{b}}\times \mathrm{Dc}\left(m,n,{\tilde{X}}_{a}\right)}{\sqrt{\{{\mathrm{Dr}\left(m,n,{\tilde{X}}_{a}\right)\}}^{2}+{\left\{\mathrm{Dr}\left(m,n,{\tilde{X}}_{a}\right)\right\}}^{2}+10^{-6}}}$$

where $\mathrm{Dr}\left(m,n,{\tilde{X}}_{\left(i,j\right)}^{\left(K\right)}\right)$ and $\mathrm{Dc}\left(m,n,{\tilde{X}}_{\left(i,j\right)}^{\left(K\right)}\right)$ are, respectively, image row and column difference operation functions.

Since we adopt the SR reconstruction method of BCS, when performing row–column direction difference operation, it will involve the cross-operation of the edge elements between adjacent image blocks. Through derivation, the difference operation between adjacent image blocks can be represented by a periodic sparse dictionary matrix, which greatly speeds up the operation speed while ensuring the accuracy. Let θ be a cyclic matrix with a period of S, i.e., θ(m,n)=θ(m+S,n)=θ(m,n+S), and its matrix structure is as follows:

(39) $$\theta =\left(\begin{array}{cccc} 1 & 2 & \cdots & S\\ S+1 & S+2 & \cdots & 2S\\ \vdots & \vdots & \ddots & \vdots \\ S^{2}-S+1 & S^{2}-S+2 & \cdots & S^{2} \end{array}\right)$$

where k is the value corresponding to the m-th row and n-th column in the matrix θ, denoted as θ(m,n); and $D_{\theta \left(m,n\right)}$ represents the row vector corresponding to the k-th row of the sparse dictionary D. Then, each HR image block $X_{b\left(i,j\right)}$ can be represented by its corresponding sparse coefficient as follows:

$$X_{b\left(i,j\right)}=\left(\begin{array}{cccc} D_{\theta \left(1,1\right)}{\tilde{X}}_{\left(i,j\right)} & D_{\theta \left(1,2\right)}{\tilde{X}}_{\left(i,j\right)} & \cdots & D_{\theta \left(1,S\right)}{\tilde{X}}_{\left(i,j\right)}\\ D_{\theta \left(2,1\right)}{\tilde{X}}_{\left(i,j\right)} & D_{\theta \left(2,2\right)}{\tilde{X}}_{\left(i,j\right)} & \cdots & D_{\theta \left(2,S\right)}{\tilde{X}}_{\left(i,j\right)}\\ \vdots & \vdots & \ddots & \vdots \\ D_{\theta \left(S,1\right)}{\tilde{X}}_{\left(i,j\right)} & D_{\theta \left(S,2\right)}{\tilde{X}}_{\left(i,j\right)} & \cdots & D_{\theta \left(S,S\right)}{\tilde{X}}_{\left(i,j\right)} \end{array}\right)$$

Then, the calculation methods of $\mathrm{Dr}\left(m,n,{\tilde{X}}_{\left(i,j\right)}^{\left(K\right)}\right)$ and $\mathrm{Dr}\left(m,n,{\tilde{X}}_{\left(i,j\right)}^{\left(K\right)}\right)$ are as follows:

$$\{\begin{array}{c} \mathrm{Dr}\left(m,n,{\tilde{X}}_{\left(i,j\right)}^{\left(K\right)}\right)=D_{\theta \left(m,n\right)}{\tilde{X}}_{\left(i,j\right)}^{\left(K\right)}-D_{\theta \left(m-1,n\right)}{\tilde{X}}_{\left(i-\neg \left(m-1\right),j\right)}^{\left(K\right)}\\ \mathrm{Dc}\left(m,n,{\tilde{X}}_{\left(i,j\right)}^{\left(K\right)}\right)=D_{\theta \left(m,n\right)}{\tilde{X}}_{\left(i,j\right)}^{\left(K\right)}-D_{\theta \left(m,n-1\right)}{\tilde{X}}_{\left(i,j-\neg \left(n-1\right)\right)}^{\left(K\right)} \end{array}$$

where ¬ is the negation operator, and the operation method is $\neg \left(z\right)=\{\begin{array}{c} 0\;z\neq 0\\ 1\;z=0 \end{array}$.

As for $g_{2}\left({\tilde{X}}^{\left(K\right)}\right)$, $\|{\tilde{X}}_{\left(i,j\right)}^{\left(K\right)}{\|}_{1}$ is a nondifferentiable convex function about ${\tilde{X}}_{\left(i,j\right)}^{\left(K\right)}$, and $\|y_{\left(i,j\right)}-CHD{\tilde{X}}_{\left(i,j\right)}^{\left(K\right)}{\|}_{2}^{2}$ is a differentiable convex function about ${\tilde{X}}_{\left(i,j\right)}^{\left(K\right)}$. Therefore, $g_{2}\left({\tilde{X}}^{\left(K\right)}\right)$ cannot be directly calculated by the gradient descent method, so it is solved by the proximal gradient (PG) method:

(40) $$\{\begin{array}{l} Z_{\left(i,j\right)}^{\left(K\right)}={\tilde{X}}_{\left(i,j\right)}^{\left(K\right)}-\beta_{\left(i,j\right)}^{\left(K\right)}D^{T}H^{T}C^{T}\left(y_{\left(i,j\right)}-CHD{\tilde{X}}_{\left(i,j\right)}^{\left(K\right)}\right)\\ {\tilde{X}}_{\left(i,j\right)}^{\left(K+1\right)}=\mathrm{Shrink}\left(Z_{\left(i,j\right)}^{\left(K\right)},\beta_{\left(i,j\right)}^{\left(K\right)}\right)=\max\{\left|Z_{\left(i,j\right)}^{\left(K\right)}\right|-\beta_{\left(i,j\right)}^{\left(K\right)},0\}\circ \mathrm{sign}\left(Z_{\left(i,j\right)}^{\left(K\right)}\right) \end{array}$$

where Shrink(·) is the soft threshold shrinkage function; $\beta_{\left(i,j\right)}^{\left(K\right)}$ essentially acts as a step factor, and its size can be determined by the backtracking line search method [32].

**Figure 3:**
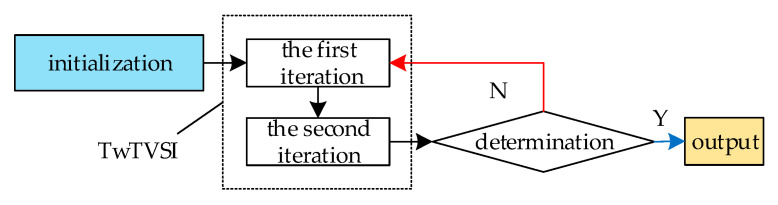
Block diagram of TwTVSI algorithm.

In summary, the two-step total variation sparse iteration algorithm flow is shown in Figure 3.

The specific calculation method of each step is as follows:

1. Initialize K and $\tilde{X}$ separately
2. Perform the first iteration of the TV constraint: solve for ${\tilde{X}}^{\left(k,1\right)}$ using Gradient descent,$\;{\tilde{X}}^{\left(k,1\right)}={\tilde{X}}^{\left(K,0\right)}-\lambda \mu^{\left(K\right)}g_{1}\left({\tilde{X}}^{\left(K,0\right)}\right)$.
3. Perform the second iteration of the Sparse constraint; according to (40), obtain ${\tilde{X}}^{\left(K+1,0\right)}$ by the proximal gradient method through ${\tilde{X}}^{\left(k,1\right)}$.
4. Determination: stop iteration if $\|Ar\left(D{\tilde{X}}^{\left(K+1,0\right)}\right)-Ar{\left(D{\tilde{X}}^{\left(K,0\right)}\right)\|}_{2}^{2}$ is less than the error constraint ε, or K is greater than the maximum number of iterations $K_{max}$. Otherwise, let K=K+1 and return to step 2.
5. Output: reconstructed HR image $Ar\left(D{\tilde{X}}^{\left(K+1,0\right)}\right)$.

Our proposed algorithm introduces the TV regular term into the $L_{1}$ norm reconstruction objective function and then uses the gradient descent method and the proximal gradient method in the optimization process to minimize the total variation of the image and solve the image sparse coding; therefore, the algorithm is named two-step total variation sparse iteration (TwTVSI).

## 5. Experiment and Result Analysis 

### 5.1. Experimental Data and Evaluation Parameters

Our test environment parameters were as follows: Intel(R) Core(TM)i5-9300H CPU @2.40GHz; memory: 16.00GB; operating system: Windows 10; MATLAB R2019a. We obtained the following fixed parameters through repeated experiments and adjustments: λ=μ=0.004; $\varepsilon_{0}=0.008$; γ=δ=2; θ=0.8; τ=0.95;ν=0.1. The image block size used for the dark channel search was 35 × 35. The sparse base Ψ used the Daubechies 8 wavelet base. The HR image block size was 32 × 32. The sparse dictionary was trained using the high-dimensional dictionary training method introduced in [34], and its size was 1024 × 3096, i.e., the dictionary contained 3096 atoms with a length of 1024.

In order to make the experimental results more convincing, we separately reconstructed artificially synthesized infrared images of power equipment and actual infrared images of power equipment. We compared our method with the methods proposed by Keys [35], Shao [19], Michaeli [17], and Kim [21]. Since the artificially synthesized infrared image of the power equipment had the original clear, HR image, the PSNR and SSIM evaluation indicators could be used to evaluate the reconstruction results. The actual infrared image of the power equipment did not have the original clear, HR image, so we adopted two other objective evaluation indicators: average gradient (AG) and information entropy (IE). The calculation method of AG is as follows:

(41) $$AG=\frac{1}{MN}\underset{i}{\sum }\underset{j}{\sum }\sqrt{f_{x}^{2}\left(i,j\right)+f_{y}^{2}\left(i,j\right)}$$

where $f_{x}\left(i,j\right)$ and $f_{y}\left(i,j\right)$ are the image convolution results of the difference operator in the row and column directions, respectively. The larger the AG value is, the more drastically the grayscale changes in the image and the more the image levels; that is, the clearer the image is.

Entropy represents the uniformity of a system in physics. The more uniformly a system is distributed, the greater its information entropy is. The concept of image information entropy is derived from this, which can be defined as follows:

(42) $$IE=-\overset{n}{\underset{i=0}{\sum }}p\left(i\right)\log_{2}p\left(i\right)$$

where p(i) represents the frequency of the pixel point with the gray value of i in the image. The larger the IE value is, the richer the information contained in the image is.

To verify the effectiveness of our proposed $L_{w}$ norm in improving the accuracy of the blur kernel estimation, we compared the blur kernels estimated by our method and the algorithms proposed in [19,21]. We used the sum of the squared differences error (SSDE) to evaluate the accuracy of the estimated blur kernel:

(43) $$SSDE=\underset{i}{\sum }\underset{j}{\sum }{\left(k_{est}\left(i,j\right)-k_{gt}\left(i,j\right)\right)}^{2}$$

where $k_{est}$ represents the estimated blur kernel and $k_{gt}$ represents the true blur kernel of the image.

In addition, we did not directly process the infrared image in RGB mode, but rather converted it to YCbCr mode, where Y is the brightness component, Cb is the blue chroma component, and Cr is the red chroma component. Since the human eye is more sensitive to the Y component, the visual difference caused by the subtle changes in the other two components is extremely small; thus, all operations of our proposed method and comparison method were performed on the Y component. Using the blur kernel estimated by the Y component, the Cb and Cr components were reconstructed by nonblind deblurring [36] and cubic interpolation.

### 5.2. Synthetic Infrared Image Reconstruction Experiment

We selected 11 different infrared images of power equipment and artificially synthesized them into LR blurred images. Below, we show the HR images reconstructed using different methods for one of the LR blurred images. For the remaining 10 images, we give the reconstruction results of different methods in the form of evaluation parameters.

**Figure 4:**
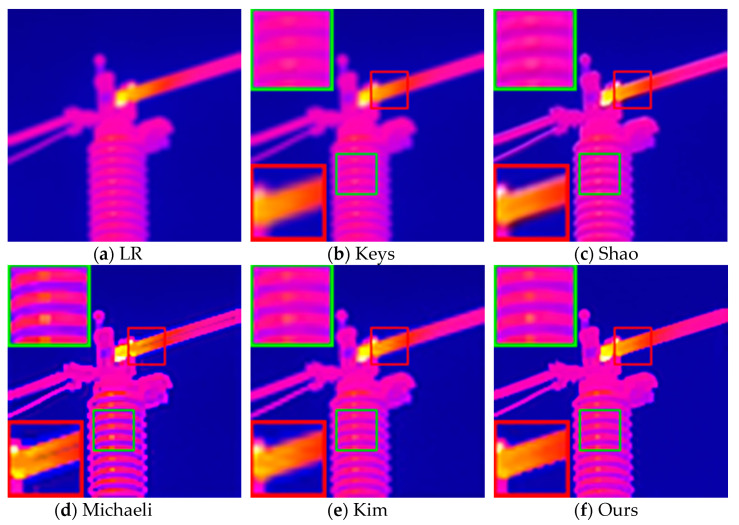
Reconstruction results of different methods when using 5 × 5 Gaussian blur kernel: (**a**) synthetic infrared image; (**b**) reconstruction results using the method in [35] (PSNR = 24.646dB; SSIM = 0.903; AG = 21.612; IE = 5.881); (**c**) reconstruction results using the method in [19] (PSNR = 25.747dB; SSIM = 0.963; AG = 27.710; IE = 5.903); (**d**) reconstruction results using the method in [17] (PSNR = 26.238dB; SSIM = 0.958; AG = 30.281; IE = 5.928); (**e**) reconstruction results using the method in [21] (PSNR = 25.321dB; SSIM = 0.955; AG = 29.591; IE = 6.014); (**f**) reconstruction results using our method (PSNR = 28.803dB; SSIM = 0.968; AG = 32.030; IE = 6.082).

A Gaussian blur kernel with a size of 5×5 and a standard deviation σ of 1.3 was used to blur the 128×128 HR infrared image, and then, it was downsampled to 64×64 through the cubic interpolation method. The original clear image, the reconstruction result of the contrast method, and the reconstruction result of our proposed method are shown in Figure 4.

As shown in Figure 4, the method proposed by Keys et al. has the worst visual effect due to the inherent smoothness benefits of interpolation algorithms, and the texture of the electrical equipment in the reconstructed image is almost invisible. Compared with the method of Shao et al., the infrared image reconstructed by the method proposed by Kim et al. has clearer edges and better visual effects, but its artifacts are relatively more obvious. It is obvious that the quality of the reconstructed image texture of the above methods is not as clear as the reconstruction result obtained by our method. The reconstructed image using Michaeli et al.’s method has a higher contrast and is too sharp. Although the edge texture is clearer than that from our method, the method introduces obvious artifacts according to the area in the red box. In addition, the color distortion of the equipment is more serious, which will have a very negative influence on the infrared diagnosis, and it can cause the operation and maintenance personnel to misjudge the equipment operating temperature.

**Figure 5:**
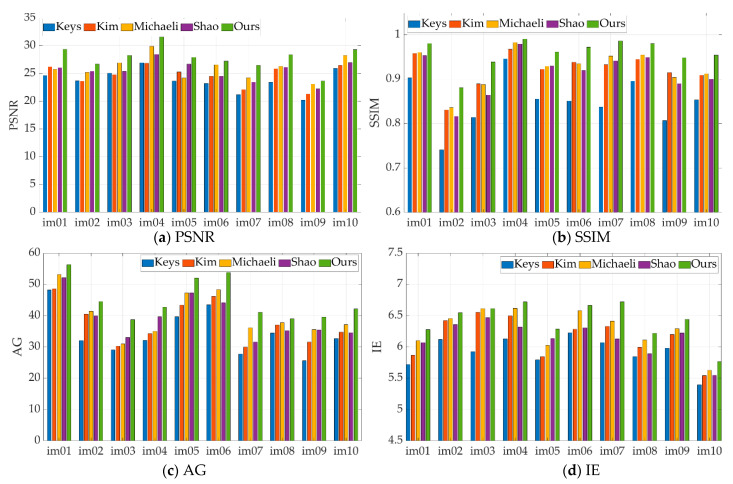
Using 5×5 Gaussian blur kernel, PSNR, SSIM, AG and IE parameter values of reconstruction results of different methods: (**a**) PSNR value; (**b**) SSIM value; (**c**) AG value; (**d**) IE value.

The remaining 10 infrared images were also artificially synthesized using the 5×5 blur kernel. The PSNR, SSIM, AG and IE values of the images reconstructed using different methods are given in Figure 5. It can be seen that the method proposed by Keys has the lowest value of various indicators, which is caused by the inherent smoothing benefits of interpolation algorithms; The evaluation parameter values of the methods proposed by Shao and Kim are often close, which is consistent with the visual quality of the reconstructed image with their method. The image color reconstructed by the Michaeli method has a certain degree of distortion, but the detailed texture and overall outline structure in the image are clearer than other comparison methods, so its method has achieved better evaluation indicators. However, our proposed method has obvious advantages compared with the contrast method in the evaluation index of most reconstructed images, which also confirms the subjective visual effect of the reconstructed by our method, and demonstrates the superiority of our proposed method.

### 5.3. Actual Infrared Image Reconstruction Experiment

**Figure 6:**
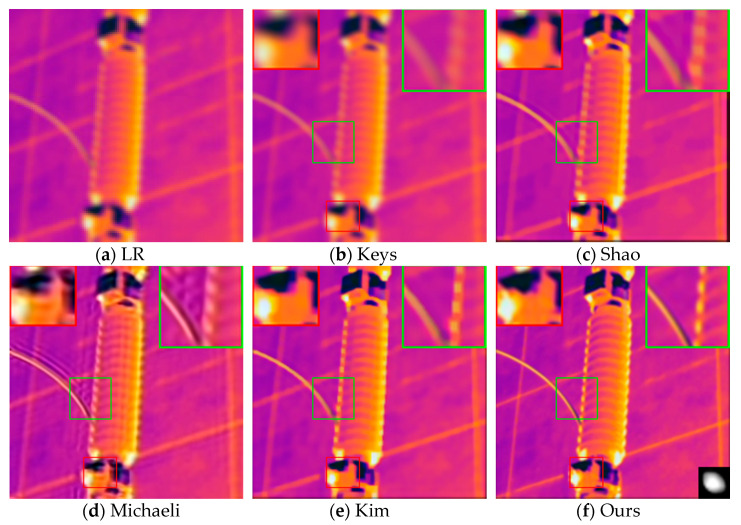
Different methods to reconstruct the real infrared image of No. 9: (**a**) real infrared image; (**b**) reconstruction results using the method in [35] (AG = 20.866; IE = 6.020); (**c**) reconstruction results using the method in [19] (AG = 29.275; IE = 6.212); (**d**) reconstruction results using the method in [17] (AG = 33.083; IE = 6.157); (**e**) reconstruction results using the method in [21] (AG = 30.449; IE = 6.188); (**f**) reconstruction results using our method (AG = 33.697; IE = 6.244).

Section 5.2 preliminarily verifies the performance of our proposed blind SR method through synthesized infrared images. In this section, we reconstruct an actual LR infrared image to verify the effectiveness of the method in practical applications. For the experiment, we used nine infrared images taken on site with a resolution of 128 × 128 for SR reconstruction. Figure 6 shows the images reconstructed using different methods for the ninth LR infrared image.

It can be seen from Figure 6 that the images reconstructed using the methods of Keys and Shao are blurry, and their details and textures are not clear. The method proposed by Michaeli et al. is not accurate enough to estimate the blur kernel, which leads to the obvious ringing effect in the reconstruction result. The obvious ringing effect is not conducive to the application of infrared diagnostic technology and will easily lead to incorrect segmentation of the equipment area, thereby affecting the accuracy of fault location and identification of fault types. The method proposed by Kim et al. has a better reconstruction effect with a clearer texture and no ringing artifacts. However, the reconstructed image is still not as clear as the result obtained by our method, which can clearly be seen from the comparison of Figure 6e,f. In summary, our method has certain advantages compared with current methods when dealing with real blurred LR images. In addition, by observing the blur kernel estimated in the reconstruction process of our method, we found that the blurring of the image is very complicated during actual shooting. Using the standard Gaussian blur kernel to represent the image blurring process is not accurate. This also reflects the necessity of studying blind SR algorithms. The SR reconstruction results of the other eight infrared images with different methods are given in Table 1 and Table 2 in the form of AG and IE indicators. It can be seen from the data in the tables that the HR image reconstructed by our method has certain advantages over the comparison method in these two indicators.

### 5.4. $L_{w}$ Norm Validity Verification

**Figure 7:**
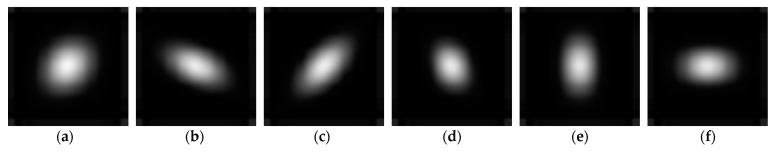
Six different blur kernels: (**a**) BK1; (**b**) BK2; (**c**) BK3; (**d**) BK4; (**e**) BK5; (**f**) BK6.

In order to verify the effectiveness of the $L_{w}$ norm constraint in improving the accuracy of the blur kernel estimation, we used the six blur kernels shown in Figure 7 to sequentially blur 100 infrared images and perform double downsampling. We used our method and the comparison methods to estimate the blur kernel based on the LR blurred image. For each blur kernel, the average SSDE parameters of the blur kernel estimated by the different methods on 100 synthetic blurred infrared images are shown in Table 3. It can be seen from the data in Table 3 that using the $L_{w}$ norm constraint improved the effectiveness of the blur kernel estimation accuracy.

### 5.5. Validity Verification of Blur Matrix

**Figure 8:**
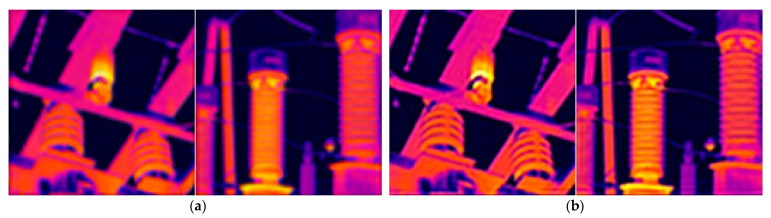
The influence of blur matrix on reconstruction effect: (**a**) infrared images reconstructed by the traditional CS method without a blur matrix; (**b**) infrared images reconstructed by our method of introducing the blur matrix into the reconstruction model.

When performing SR modeling based on the CS theory, we introduced a blur matrix to deconvolute and deblur the reconstructed image. Figure 8 is a comparison diagram of the reconstruction results with and without the introduction of the blur matrix in our model. It can be clearly seen from Figure 8 that when no blur matrix is added to the reconstruction model, the reconstructed image is blurry. When the blur matrix is added, the visual effect of the reconstructed image is significantly improved, the edge contrast is higher, and the outline is clearer. It can be concluded that the addition of a blur matrix is beneficial to the improvement of image reconstruction quality.

### 5.6. Analysis of TwTVSI Algorithm Performance

**Figure 9:**
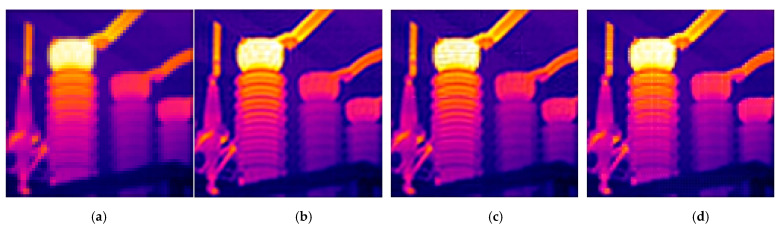
Reconstruction results of different block compressed sensing algorithms: (**a**) LR image; (**b**) image reconstructed by TwTVSI algorithm; (**c**) image reconstructed by BCS-L1 algorithm; (**d**) image reconstructed by BCS-TV algorithm.

In this section, we analyze the effect and computing time of the TwTVSI algorithm to verify the effectiveness of the improved model and the designed algorithm. Figure 9 shows the comparison of the reconstruction results of our algorithm, the BCS-L1 algorithm with only a sparse constraint in the objective function, and the BCS-TV algorithm with only a TV constraint in the objective function. It can be seen from Figure 9 that the HR image reconstructed by the BCS-L1 algorithm has an obvious “blocking effect” due to BCS; that is, the boundary between image blocks is obvious and discontinuous. Although there is no “blocking effect” in the image reconstructed using the BCS-TV algorithm, the algorithm only restricts the sparsity of the image gradient domain and cannot make good use of the sparsity of other change domains of the image. As a result, bright spots appear in the reconstruction results, and the reconstruction speed is too slow. Table 4 shows the calculation time required by the TwTVSI algorithm, BCS-L1 algorithm, and BCS-TV algorithm to reconstruct five LR images under the same experimental conditions. It can be seen that the reconstruction speed of the TwTVSI algorithm is slightly slower than that of the BCS-L1 algorithm, but both are of the order of 10 s, while the reconstruction time of the BCS-TV algorithm is on the order of 1000 s.

## 6. Discussion on Future Application Scenarios

At present, infrared images are still generally processed in the central place of control, but now the emergence of 5G communications, cloud computing, cloud platforms, distributed computing and other technologies provides many new possibilities for the processing and analysis of infrared images in the future. For example, it is no longer limited to processing infrared images at a specific location, the raw data can be uploaded to the cloud platform through 5G communication, and then call the computing power through cloud computing to complete the image processing. Alternatively, we can use distributed computing, call on local idle computing power, complete the analysis and processing of the image, and upload the final evaluation results and original data. The evaluation results are uploaded to the operation and maintenance platform for operation and maintenance personnel to read, and the original data is directly stored in the database for future data analysis, which realizes the further distribution of functions and reduces the platform’s data processing and interaction pressure. After providing the cloud platform access port for operation and maintenance personnel, they can accurately grasp the operating status of the equipment through mobile phones and computers at any time and any place, and then complete the operation and maintenance work according to the platform’s prompts.

## 7. Conclusions

Infrared thermal imaging technology is an important technical means to ensure the safe and reliable operation of power equipment, through which faults in power equipment can be detected in time. In order to resolve the problem of insufficient resolution and definition of infrared images, we proposed a blind SR algorithm based on the theory of CS.

In order to complete the blind SR, the image blur kernel needed to be estimated first. We combined the basic CS SR model with the blind image deblurring theory and then proposed a blur kernel estimation algorithm under the CS framework to realize the estimation of the blur kernel from LR images. In the estimation process, we defined a new $L_{w}$ norm to constrain the gradient image in the iterative process by analyzing the changes in the significant edge intensity before and after the image is blurred. The $L_{w}$ norm constraint can select and enhance the salient edges, make the intermediate latent image generated by the iteration move closer to the clear image, and improve the accuracy of the blur kernel estimation. Experimental results proved that the blur kernel estimation algorithm we proposed can effectively and accurately estimate the corresponding blur kernel based on LR infrared images. Our improvement to the edge selection method is also conducive to improving the accuracy of blur kernel estimation results. 

In the SR image reconstruction part, we added a blur matrix to the original CS model, thereby constructing a CS super-resolution model based on the principle of image degradation. The sparse base in the model is a specific sparse dictionary about the infrared image of the power equipment, which was obtained using the high-dimensional dictionary training method. The sparse dictionary can enhance the sparse representation of the image and improve the SR reconstruction effect. Finally, according to the theory of BCS, we designed the TwTVSI algorithm, which can effectively process the CS super-resolution model with the sparse dictionary. TwTVSI also can eliminate the “blocking effect” so as to complete high-quality SR reconstruction of infrared images. The SR reconstruction experiments with blurred LR infrared images verified the effectiveness of the blind SR method that we proposed. The experiments proved that our method can better adapt to the actual application in and demand of the electric power industry. Our method is helpful in allowing operation and maintenance personnel to accurately grasp the operating status of power equipment and for improving the reliability of power system operation.

## Figures and Tables

**Table 1:** Comparison of AG indexes of images reconstructed using different methods.

Image Number	Keys	Shao	Michaeli	Kim	Ours
1	19.875	27.031	30.784	29.339	32.364
2	22.098	29.508	34.180	32.142	35.882
3	21.671	29.874	34.954	35.377	37.047
4	18.124	20.616	23.167	25.423	24.267
5	18.664	26.541	29.313	28.800	30.954
6	17.381	25.453	26.271	26.299	28.534
7	24.336	34.171	35.325	37.638	42.721
8	20.385	26.413	34.928	27.116	32.957

**Table 2:** Comparison of IE indexes of images reconstructed using different methods.

Image Number	Keys	Shao	Michaeli	Kim	Ours
1	5.904	6.103	6.111	6.148	6.275
2	6.577	6.413	6.657	6.709	6.748
3	6.218	6.281	6.338	6.342	6.381
4	5.730	5.803	5.862	5.881	5.906
5	6.133	6.141	6.247	6.243	6.298
6	5.605	5.571	5.660	5.691	5.772
7	6.722	6.364	6.765	6.772	6.846
8	5.813	5.852	5.890	5.883	5.916

**Table 3:** The mean SSDE of each method for each BK on all 100 synthetic blurred images.

Image Number	Shao	Michaeli	CS-L0 ^1^	Ours
BK1	0.0473	0.0485	0.0481	0.0461
BK2	0.0472	0.0438	0.0453	0.0420
BK3	0.0467	0.0444	0.0461	0.0422
BK4	0.0390	0.0379	0.0368	0.0353
BK5	0.0431	0.0429	0.0435	0.0426
BK6	0.0422	0.0406	0.0412	0.0393

^1^ “CS-L0” refers to the blur kernel estimation method based on the principle of CS in Section 3.1, but the $L_{0}$ norm is used to constrain the gradient image.

**Table 4:** Reconstruction time of each algorithm.

Image Number	TwTVSI (s)	BCS-L1 (s)	BCS-TV (s)
1	15.3293	6.5937	1214.3894
2	14.2397	5.4419	1175.4316
3	14.8195	6.2824	1135.6211
4	15.4857	6.6621	1308.4803
5	15.0018	5.7140	1260.2702

## Data Availability

Not applicable.
